# Accelerated soil carbon turnover under tree plantations limits soil carbon storage

**DOI:** 10.1038/srep19693

**Published:** 2016-01-25

**Authors:** Guangshui Chen, Yusheng Yang, Zhijie Yang, Jinsheng Xie, Jianfen Guo, Ren Gao, Yunfeng Yin, David Robinson

**Affiliations:** 1 Key Laboratory for Subtropical Mountain Ecology (Ministry of Science and Technology and Fujian Province Funded), School of Geographical Sciences, Fujian Normal University, Fuzhou 350007, China; 2 Institute of Biological & Environmental Sciences, School of Biological Sciences, University of Aberdeen, Aberdeen AB24 3UU, UK

## Abstract

The replacement of native forests by tree plantations is increasingly common globally, especially in tropical and subtropical areas. Improving our understanding of the long-term effects of this replacement on soil organic carbon (SOC) remains paramount for effectively managing ecosystems to mitigate anthropogenic carbon emissions. Meta-analyses imply that native forest replacement usually reduces SOC stocks and may switch the forest from a net sink to a net source of atmospheric carbon. Using a long-term chronosequence during which areas of subtropical native forest were replaced by Chinese fir, we show by direct measurement that plantations have significantly accelerated SOC turnover compared with native forest, an effect that has persisted for almost a century. The immediate stimulation of SOC decomposition was caused by warmer soil before the closure of the plantation’s canopy. Long-term reductions in SOC mean residence times were coupled to litter inputs. Faster SOC decomposition was associated with lower soil microbial carbon use efficiency, which was due to smaller litter inputs and reduced nutrient availabilities. Our results indicate a previously unelucidated control on long-term SOC dynamics in native forests and demonstrate a potential constraint on climate mitigation when such forests are replaced by plantations.

Four million hectares of global primary forests have been lost annually since 1990, mostly in tropical and subtropical areas. This loss has coincided with an annual 2% increase in the total area of tree plantations, which now cover over a quarter of a billion hectares of land^1^. Soils of native old-growth forests have a large carbon (C) storage capacity and can act as net sinks for atmospheric CO_2_^2^. Soil organic carbon (SOC) comprises three-fourths of all terrestrial C and is a long-term buffer against atmospheric CO_2_ increases^3^,^4^,^5^. This important role of native forests in terrestrial C storage raises concerns about long-term consequences of their replacement by plantations, though plantations have been advocated as a measure to sequestrate C from the atmosphere and to mitigate future climate change^6^.

Global-scale meta-analyses^7^,^8^ suggested that replacement of native forests by tree plantations has generally reduced SOC stocks by 13–19%, the effect being most serious in the first two decades, although in the tropics, conversion of native forests to plantations has had no significant effect on SOC^9^. Meta-analyses necessarily include studies spanning diverse sites and species to capture statistical patterns that single studies cannot, but with an acknowledged risk that the mechanisms underlying those patterns can sometimes be obscured^9^. An additional problem with the soil C data available for meta-analysis is that most measurements are restricted to the first two decades of forest regrowth^8^; data covering all stages of plantation development are scarce. This hinders our ability to reliably predict the long-term dynamics of soil C during reforestation and to understand their controlling mechanisms.

Mechanistic understanding of the processes regulating soil C storage requires direct measurements of belowground C pools and fluxes. An ecosystem’s SOC stock declines if SOC losses exceed inputs. Inputs are those from vegetation (litter, dead roots) and organic C exuded from roots. Losses occur as mainly respiratory CO_2_ produced by heterotrophic microbes that decompose SOC, autotrophic respiration, and smaller C fluxes as methane and volatile organics. C inputs generally decrease following conversion of natural forests to plantations: on average, aboveground litterfall and fine-root biomass are respectively 34% and 66% smaller in plantations than in natural forests^8^. Thus, decreased plant C input could be an important driver of SOC reduction during reforestation. However, the extent to which that reduction is caused by accelerated decomposition of SOC is still unknown.

*In situ* measurements of the CO_2_ flux from SOC decomposition (*R*_*m*_) are difficult since the CO_2_ produced by that process is usually mixed with fluxes from other pathways, particularly litter decomposition (*R*_*l*_) and autotrophic respiration by roots and symbionts (*R*_*r*_). The sum of these three fluxes constitutes the total soil-surface CO_2_ flux (*R*_*s*_)^10^,^11^. Most studies of soil CO_2_ production report only *R*_*s*_, but there is an increasing need to separate *R*_*s*_ into its components to determine the distinct constraints on different pathways of SOC loss^12^.

In this study, we used a chronosequence of Chinese fir plantations established by successively clearing mixed-species native forests over 88 years (Supplementary Table S1)^13^. As an important timber species, Chinese fir covers over 12 Mha, almost 11% of the global planted forest area^1^. We established experimental plots that allowed *R*_*s*_, *R*_*h*_ and *R*_*m*_ to be measured and *R*_*l*_ derived, and from which SOC residence times and functional aspects of soil microbial activity were estimated. We were therefore able to directly test long-term effects of replacing native forests by plantations on processes controlling the size and dynamics of the SOC pool.

## Results

### Changes in soil temperature and moisture across the chronosequence

**Figure 1:**
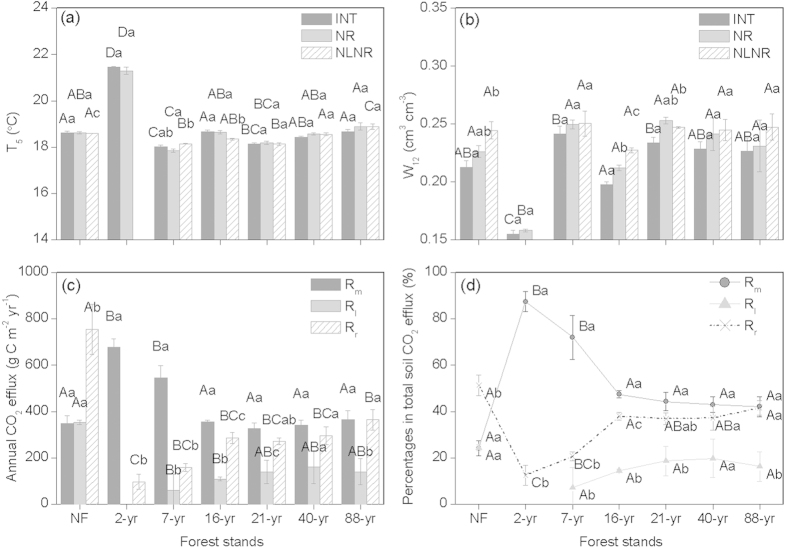
Annual mean soil environmental factors and annual component fluxes of soil respiration across the Chinese fir chronosequence in southern China (from 2- to 88-year-old, native forest (NF) as the control). (**a**) Soil temperature at 5 cm depth (T_5_); (**b**) soil moisture at the 0-12 cm depth (W_12_) for the intact (INT), root exclusion (NR), and litter plus root exclusion (NLNR) treatments; and (**c**) annual CO_2_ effluxes from decomposition of mineral SOC (*R*_*m*_), litter decomposition (*R*_*l*_) and root and rhizosphere respiration (*R*_*r*_), and (**d**) their contributions to total annual soil CO_2_ effluxes. The error bars represent standard errors [n = 3 (plots)]. Data with the same upper-case letters are not significantly different among stands and those with the same lower-case letters are not significantly different among treatments (*p* > 0.05).

Plantation establishment caused only a transient increase in soil temperature in the first two years following planting, before full canopy closure. Following the removal of the native forest’s canopy, mean soil temperature at 5 cm depth became about 3 ^o^C warmer (Fig. 1a; Supplementary Fig. S1). Development of the Chinese fir canopy within 7 yr of planting restored the soil temperature to that of the native forest. Establishment of the experimental plots generally had no effect on soil temperature (Fig. 1a; Supplementary Fig. S1). Temperature changes were mirrored by changes in soil moisture. Two years after clearance, the soil was drier compared with under native forest, but wetter once the plantation had developed (Fig. 1b; Supplementary Fig. S2). Slightly wetter soil was associated with trenched plots, which is expected because of the absence of the drying effect of transpiration^14^.

### Changes in soil respiration and its partitioning across the chronosequence

Rates of soil-surface CO_2_ efflux (*R*_*s*_) generally followed the seasonal patterns of soil temperature (Supplementary Fig. S3), with high rates during summer (from June to August) and low rates during winter (From December to February). *R*_*s*_ varied significantly by stands (*p* < 0.001), experimental plots (*p* < 0.001) and stand × experimental plots (*p* < 0.001) (Supplementary Table S2). There was a dramatic decline of *R*_*s*_ in July 2007 in some stands (e.g., the 2-year stand) (Supplementary Fig. S3), probably as a result of decreasing soil moisture during that period (Supplementary Fig. S2). *R*_*s*_ generally increased exponentially with soil temperature (Supplementary Table S3). Soil moisture varied significantly with *R*_*s*_ only for the intact plots in the native forest and in the 7-yr old stand, and the trenched plots in the 2-yr old stand (Supplementary Table S4).

Partitioning of soil respiration varied significantly between native forest and plantations, with a greater contribution to total CO_2_ flux by SOC decomposition from plantations relative to the native forest. *R*_*s*_ in native forest and plantations was 737–1457 g C m^−2^ yr^−1^, within the range expected for subtropical forests^15^,^16^. The CO_2_ flux from SOC decomposition (*R*_*m*_) in the 2- and 7-yr old stands (678 and 547 g C m^−2^ yr^−1^, respectively) significantly exceeded that in other stands, including native forest (Fig. 1c). Native forest had a higher annual CO_2_ efflux associated with litter decomposition (*R*_*l*_), 353 g C m^−2^ yr^−1^, than in the 7- and 16-yr old stands. CO_2_ efflux from rhizosphere respiration (*R*_*r*_) in native forest was 756 g C m^−2^ yr^−1^, up to 8-fold greater than in the Chinese fir stands (Fig. 1c). Translated into percentage contributions to the total soil-surface flux, these data show that in native forest, *R*_*r*_ predominated, contributing 51%, while *R*_*m*_ and *R*_*l*_ each accounted for about 25% (Fig. 1d). *R*_*m*_ contributed almost all of the soil-surface CO_2_ flux in 2- and 7-yr old plantations and persistently exceeded *R*_*m*_ in the native forest thereafter, *R*_*r*_ and *R*_*l*_ comprising less than 40% of the total (Fig. 1d).

### Changes in soil microbial characteristics across the chronosequence

**Figure 2:**
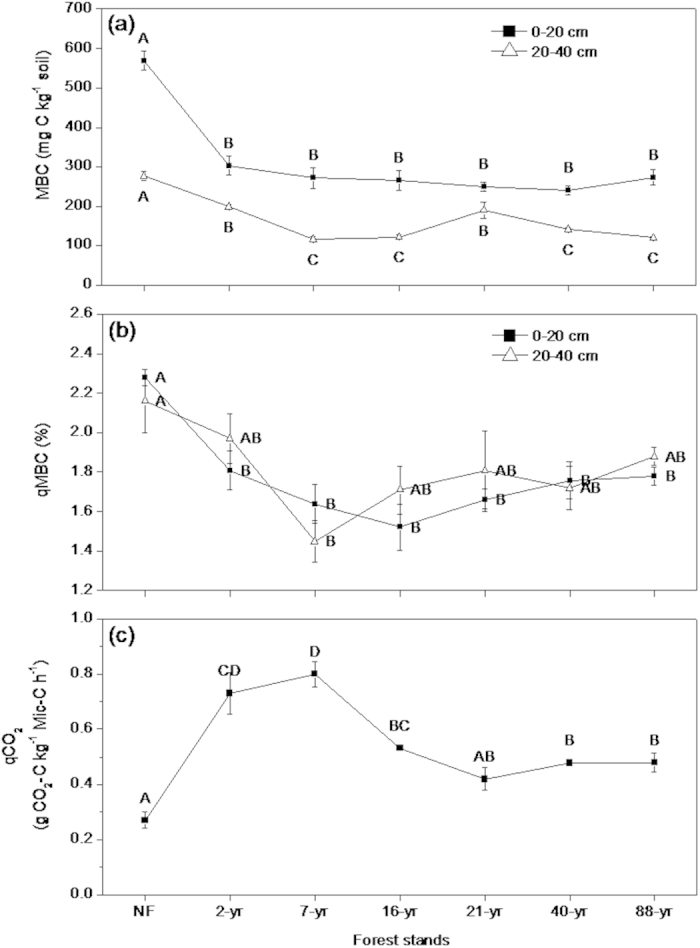
Soil microbial characteristics across the Chinese fir chronosequence in southern China (from 2- to 88-year-old, native forest (NF) as the control): (**a**) microbial C concentration (MBC), (**b**) microbial C quotient (qMBC), and (**c**) microbial metabolic quotient (qCO_2_). The error bars represent standard errors [n = 3 (plots)]. Data with the same letters are not significantly different among stands (*p* > 0.05).

Replacement of native forest by plantations caused a shift from a large, metabolically efficient microbial community to a smaller, less efficient microbial community under plantations. We found that the total microbial biomass C (MBC) in native forest soil was always greater than under Chinese fir, both near the surface and deeper in the profile (Fig. 2a). The proportion of MBC in SOC (qMBC) was 2.3% under native forest, but decreased following planting and never recovered during stand maturation (Fig. 2b). The metabolic quotient of soil microbes (qCO_2_), was minimal in native forest and trebled in the 2- and 7-yr old stands (Fig. 2c). It decreased and remained steady thereafter, but was always greater than in native forest.

### Changes in SOC turnover across the chronosequence

**Figure 3:**
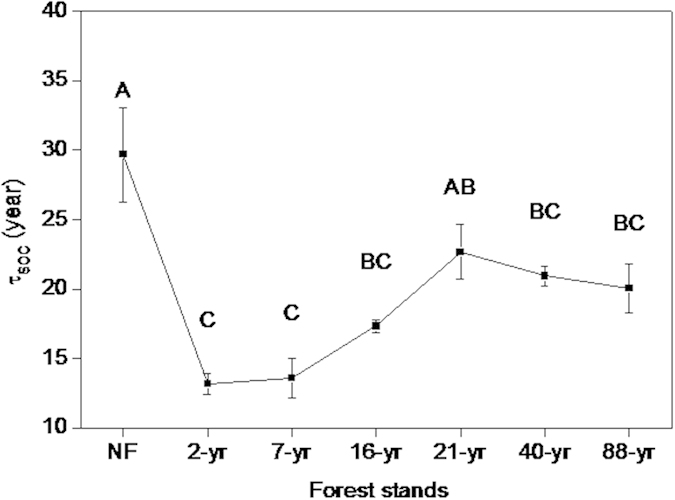
Mean residence time of SOC (τ_SOC_) across the Chinese fir chronosequence in southern China (from 2- to 88-year-old, native forest (NF) as the control). The error bars represent standard errors [n = 3 (plots)]. Data with the same letters are not significantly different among stands (*p* > 0.05).

Plantation establishment accelerated SOC turnover immediately after planting, an effect that has persisted for almost a century. SOC mean residence time (τ_SOC_) in native forest was about 30 yr, but in 2- and 7-yr old stands it decreased by over 50% (Fig. 3). Although τ_SOC_ increased with stand maturation, it remained persistently shorter under Chinese fir even in the oldest stands.

**Figure 4:**
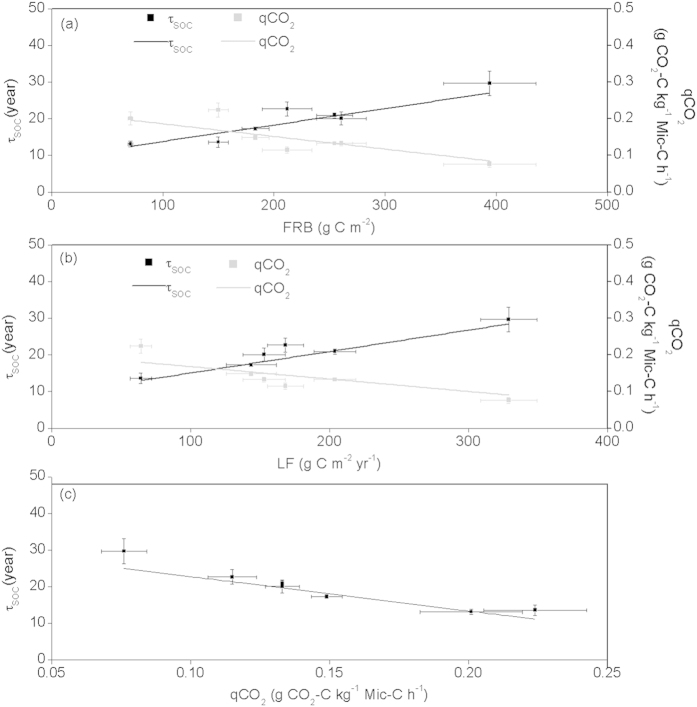
Linear regression between (**a**) fine root biomass (FRB) and mean residence time of SOC (τ_SOC_) (τ_SOC_ = 0.045FRB + 9.33, R^2^ = 0.88, *p* = 0.001, n = 7), and microbial metabolic quotient (qCO_2_) (qCO_2_ = −0.00034FRB + 0.22, R^2^ = 0.68, *p* = 0.014, n = 7); between (**b**) litterfall (LF) and τ_SOC_ (τ_SOC_ = 0.058LF + 9.24, R^2^ = 0.86, *p* = 0.005, n = 6), and qCO_2_ (qCO_2_ = −0.00034LF + 0.20, R^2^ = 0.58, *p* = 0.048, n = 6); and between (**c**) qCO_2_ and τ_SOC_ (τ_SOC_ = −30.5qCO_2_ + 35.8, R^2^ = 0.92, *p* < 0.001, n = 7). The error bars represent standard errors [n = 3 (plots)].

Long-term responses of soil C turnover were coupled to soil microbial activity and litter inputs, highlighting mechanistic links between resource availability, microbial physiology, and soil C turnover. τ_SOC_ was correlated positively with litterfall (*R*^*2*^ = 0.86, *n* = 6, *p* = 0.005) and fine-root biomass (*R*^*2*^ = 0.88, *n* = 7, *p* = 0.001) (Fig. 4a,b). Conversely, τ_SOC_ was correlated negatively with qCO_2_ (*R*^*2*^ = 0.92, *n* = 7, *p* < 0.001) (Fig. 4c). Also, qCO_2_ was correlated negatively with annual litterfall (*R*^*2*^ = 0.58, *n* = 6, *p* = 0.048), and fine-root biomass (*R*^*2*^ = 0.68, *n* = 7, *p* = 0.014) (Fig. 4a,b). These correlations indicate that larger organic C inputs are associated with more metabolically efficient soil microbes able to rapidly process labile SOC, leading to greater SOC persistence, features associated with native forests rather than plantations.

## Discussion

These analyses clarify mechanisms by which belowground C storage and dynamics are altered when mixed-species native forests are replaced by single-species tree plantations. Besides the smaller above- and belowground plant inputs, accelerated decomposition of SOC could be the important mechanism causing reduced long-term SOC storage under Chinese fir plantations compared with native forest.

The high CO_2_ flux from SOC decomposition (*R*_*m*_) and also the rapid turnover of SOC indicated by its short mean residence time in the 2-year stand reflects the higher soil temperatures that follow forest canopy removal and lack of ground cover. Weakened physical protection of SOC by soil mineral particles and soil aggregates during harvesting, burning, site preparation and management might also stimulate SOC decomposition. However, the enduring effects on SOC turnover are explained by shifts in soil microbial composition and function that are associated with forest conversion^17^,^18^,^19^,^20^,^21^.

Soil microbes are responsible for the majority of SOC decomposition and are the key regulators of SOC dynamics^22^. qCO_2_ measures the short-term partitioning of microbially assimilated C between biomass production and respiration^22^. Integrated over decadal timescales, reductions in qCO_2_ imposed by the replacement of native forest by plantations have had a profound effect on long-term soil C sequestration and soil-atmosphere CO_2_ exchange^23^.

The strong correlation between τ_SOC_ and qCO_2_ across the chronosequence indicates the pervasive influence of microbial physiology on soil C processes. The higher qCO_2_ under the Chinese fir plantations compared with natural forests, a finding that confirms previous reports^21^,^24^,^25^, could indicate changes in substrate utilization patterns by soil microbes, such as increases in maintenance respiration and decreases in microbial C use efficiency. qCO_2_ varies inversely with soil fungal:bacterial biomass ratio^26^, so the changes in soil microbial metabolism accompanying forest conversion that we found are likely to be associated with shifts in microbial community composition, especially with decreased abundance and diversity of soil fungi in tree plantations^21^,^24^.

The changes in microbial efficiency associated with forest conversion might also reflect altered nutrient status in soils. In native forests, soil microbes are not severely nutrient-limited, due to high soil nutrient availability and large annual inputs of above- and belowground litter (and nutrients therein) (Supplementary Table S1) relative to the nutrient demands of trees^27^. Soil microbes metabolize C efficiently and SOC buildup is favoured by accumulation of microbial residues^28^. Under such conditions, the effects on SOC can be dramatic: undisturbed old-growth native forest soils in subtropical China accumulate SOC at 0.54–0.68 Mg C ha^−1^ yr^−1 2^, representing a strong net sink for atmospheric CO_2_.

Reductions in soil nutrient availability are widespread following the replacement of native forests by plantations^29^, especially in tropical regions where fertilization is uncommon in tree plantations and where soil P is generally low in availability and replenished only slowly from rock weathering^30^. Across our chronosequence, available P and mineral N (NH_4_^+^–N plus NO_3_^−_^N) in soil fell from 4.71 and 16.7 mg kg^−1^, respectively, in native forest to 2.63 and 12.3 mg kg^−1^ in the 88-yr-old plantation (Supplementary Table S1). Most nutrient depletion occurred in the first 7 yr, caused initially by burning and subsequently by soil erosion^29^. The largest soil mineral N pool occurred under the 2-yr old stand, suggesting rapid stimulation of N mineralization driven by the increased soil temperature following native forest removal. This would risk substantial loss of labile soil N during that period, as the ground cover was scarce and nutrient uptake by the small seedlings limited. However, as Chinese fir trees attained maximum growth rates (between 7 yr and 21 yr old), their nutrient demands increased correspondingly while soil nutrient pools as well as litter inputs and nutrients therein^13^,^27^ were greatly reduced relative to those in native forest (Supplementary Table S1). Soil heterotrophs under plantations have less access to nutrients, metabolize C less efficiently, and must decompose more soil organic matter (SOM) to meet their nutrient demands and those of the trees. Consequently, SOC turns over rapidly and its buildup is impeded^13^. As plantations mature, SOC decomposition returns to its pre-plantation rate. But that recovery is not accompanied by a corresponding increase in τ_SOC_ and SOC storage^13^ because the nutrient demand-supply balance of planted trees cannot recover to that of native forests. Consequently, the SOC pools under even the oldest tree plantations in our study had not recovered to those of native forests^13^, and could be reduced even further in successive rotations^31^. This echoes the effect that replacement of native by non-native grasses has on SOC storage in temperate grasslands^32^, but has not previously been reported for forests. It contrasts with the widely reported conclusion from meta-analyses that SOC densities under plantations can apparently recover after about 40 years^7^.

Accelerated turnover of SOC under tree plantations contradicts previous findings that plantations usually accumulate less labile SOC in terms of dissolved organic C, MBC, light fraction organic C, and particulate organic C compared with native forests^18^,^33^,^34^,^35^. This result is consistent with the growing view^36^ that SOM stability is dominated by environmental and biological controls and not solely by its molecular complexity. Furthermore, recent field experiments have demonstrated that the identity of plantation tree species and their type of mycorrhizal symbiosis can have large yet distinct influences on SOC dynamics^37^.

Our findings have potentially serious implications for the management of long-term C storage in forest soils. Native forests have belowground C processes that promote accumulation and long-term persistence of SOC^36^; plantations do not. Ecosystem C management will be significantly jeopardized if plantations are located on land previously occupied by native forests. From a carbon management point-of-view, native forests should be preserved and alternative sites for large-scale plantations, such as abandoned cropland or reclaimed industrial areas, preferred. Management of plantations for C sequestration should integrate C and nutrient management strategies to sustain SOC formation while reducing decomposition, through optimizing nutrient conservation by avoiding soil erosion and vegetation burning, and maximizing litter inputs to soils. Efforts to promote natural secondary forest regeneration following selective harvesting rather than planting plantations on clear-cut land should also be valued to gain co-benefits of carbon storage and increased biodiversity^38^.

## Methods

### Site description

The study sites were located in a small watershed in Fujian Province, China. The dominant vegetation of the region was once evergreen broadleaved forest, but is now a mosaic of Chinese fir (*Cunninghamia lanceolata* (Lamb.) Hook.) plantations. A chronosequence of Chinese fir plantations of 2, 7, 16, 21, 40 and 88 years old, all of which were planted on natural forest land following clear-cutting and burning, was selected to represent full stand development, with a nearby native broadleaved forest as a reference (Supplementary Table S1). The understorey vegetation in all Chinese fir stands comprised mainly shrubs and herbs. The native forest was dominated by *Castanopsis fargesii*, *Schima superba* and *Pinus massoniana* and had a substantial understorey community. These forests are neighbouring (<1 km apart) and have similar parent material, soil type, and topography.

### Measurements

In January 2006, three 20 m × 20 m plots were established in each forest. Within these, replicate (*n* = 3) subplots, 0.5 m × 1.0 m, were established to provide intact controls (INT) and root exclusion (no-roots: NR) or root exclusion plus litter removal (no-roots, no-litter: NRNL) treatments. In the latter two treatments, subplots were trenched and lined with plastic to prevent new root in-growth.

Soil-surface CO_2_ flux (*R*_*s*_), along with soil temperature at 5 cm depth (T_5_) and soil moisture at 12 cm depth (W_12_), from each subplot was measured monthly from July 2006 to June 2008 using three automated infra-red gas analyzers coupled to soil-surface chambers (Li-8100, Li-Cor Inc., Lincoln, NE, USA). The sampling time was restricted between 09:00 am and 11:00 am on rainless mornings when the *R*_*s*_ rates were not significantly different from the daily means irrespective of stand and season^16^. We directly measured *R*_*s*_ (in INT plots), heterotrophic CO_2_ flux (*R*_*h*_, in NR plots) and the CO_2_ flux from SOC decomposition (*R*_*m*_, in NRNL plots) and calculated the CO_2_ fluxes from litter decomposition (*R*_*l*_ = *R*_*h*_-*R*_*m*_) and living roots and symbionts (*R*_*r*_ = *R*_*s*_-*R*_*h*_). Monthly fluxes were derived by multiplying daily fluxes by the number of days in the month, and annual fluxes computed as the sum of the monthly fluxes^39^,^40^.

The mean residence time of SOC (τ_SOC_) was estimated^41^ by dividing the SOC stock of the 0–100 cm depth by annual *R*_*m*_. Soil microbial biomass C (MBC) from the 0–20 cm and 20–40 cm soil depths was determined by the fumigation extraction method^42^. Microbial C quotient (qMBC) was calculated as the fraction of MBC present in SOC^43^. Metabolic quotients (qCO_2_) were determined by dividing field-measured *R*_*m*_ of the corresponding month when MBC was measured by the size of the MBC pool.

### Statistics

The within-stand replicated plots were considered to be the experimental units, and all the variables were averaged within each plot. A repeated measures ANOVA was performed, after checking assumptions of sphericity with Mauchley’s test, to evaluate if stand, treatment and sampling month had significant effects on *R*_*s*_ rates, T_5_ and W_12_. All comparisons among stands or among treatments were performed using a one-way ANOVA followed by Tukey’s HSD tests. Linear regression analysis was performed to examine the relationship between ln-transformed monthly soil respiration rates and T_5_. To test whether W_12_ had an effect on soil respiration, we used both T_5_ and W_12_ (or ln-transformed W_12_) as two predictors of ln-transformed soil respiration and conducted a multiple linear regression. A coefficient of W_12_ for which *p* < 0.05 indicated a statistically significant effect on soil respiration. τ_SOC_ and qCO_2_ were related to annual litter production, fine-root biomass, SOC pool size, mean annual T_5_, and mean annual W_12_ by linear regressions to determine factors affecting soil C cycling across the chronosequence. Data on litter production, fine-root biomass and SOC pool were reported previously^13^.

## Additional Information

**How to cite this article**: Chen, G. *et al.* Accelerated soil carbon turnover under tree plantations limits soil carbon storage. *Sci. Rep.* **6**, 19693; doi: 10.1038/srep19693 (2016).

## Supplementary Material

Supplementary Information
