# Supplementary material for: Accelerated soil carbon turnover under tree plantations limits soil carbon storage
Source: Sci Rep. 2016 Jan 25;6:19693. doi: 10.1038/srep19693 (PMC4726314; doi:10.1038/srep19693)
Supplement: Supplementary Information [file srep19693-s1.pdf]

Title: Accelerated soil carbon turnover under tree plantations limits soil carbon storage

Authors: Guangshui Chen[1*], Yusheng Yang[1*], Zhijie Yang[1], Jinsheng Xie[1], Jianfen Guo[1], Ren Gao[1], Yunfeng Yin[1] and David Robinson[2]

Affiliations: [1]Key Laboratory for Subtropical Mountain Ecology (Ministry of Science and Technology and Fujian Province Funded), School of Geographical Sciences, Fujian Normal University, Fuzhou 350007, China; [2]Institute of Biological & Environmental Sciences, School of Biological Sciences, University of Aberdeen, Aberdeen AB24 3UU, UK

***Author for correspondence:** Guangshui Chen (gschen@fjnu.edu.cn); Yusheng Yang (geoyys@fjnu.edu.cn)

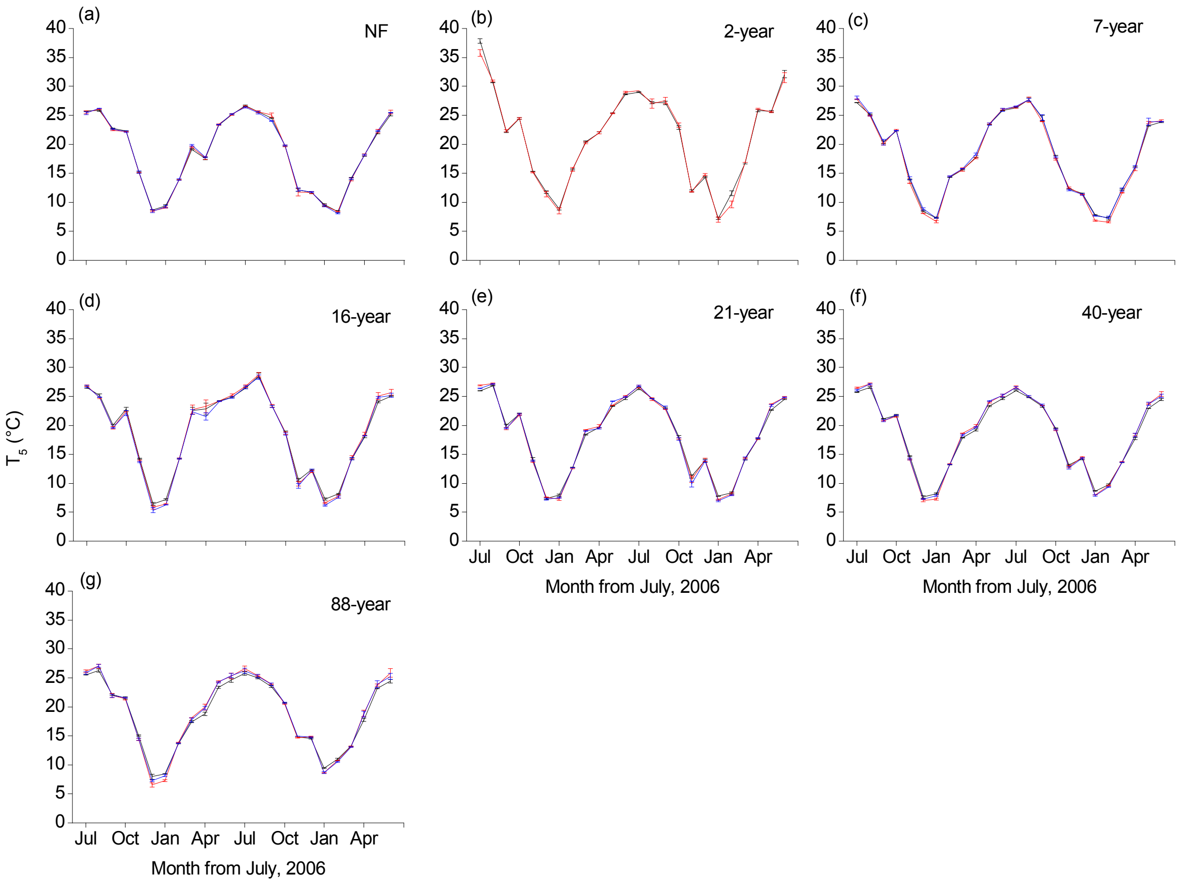

Supplementary Figure S1. Month soil temperature at the depth of 5 cm from July 2006 to June 2008 for the intact (black), root exclusion (red), and litter plus root exclusion (blue) treatments across the Chinese fir chronosequence in southern China (from 2- to 88-year-old, native forest (NF) as the control). The error bars represent standard errors [n = 3 (plots)].

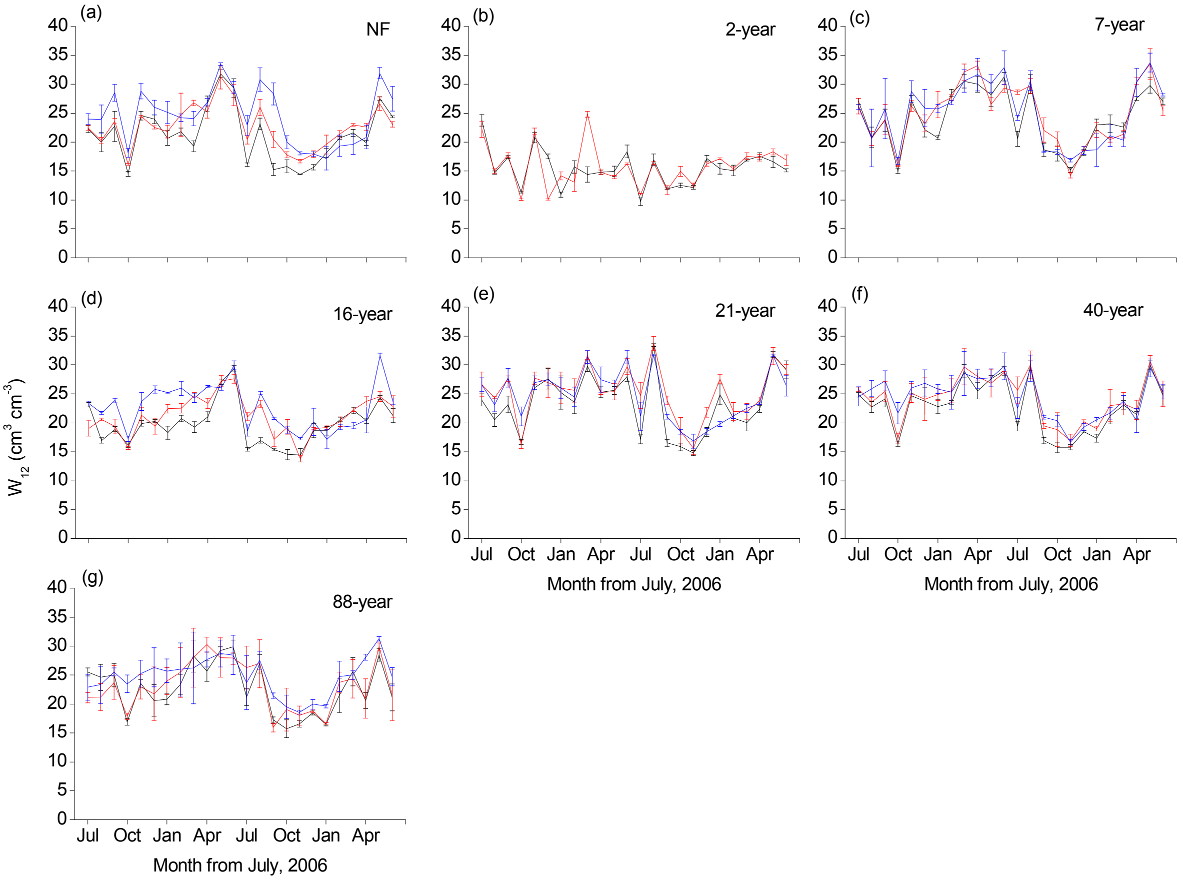

Supplementary Figure S2. Month soil moisture at the depth of 0-12 cm from July 2006 to June 2008 for the intact (black), root exclusion (red), and litter plus root exclusion (blue) treatments acroos the Chinese fir chronosequence in southern China (from 2- to 88-year-old, native forest (NF) as the control). The error bars represent standard errors [n = 3 (plots)].

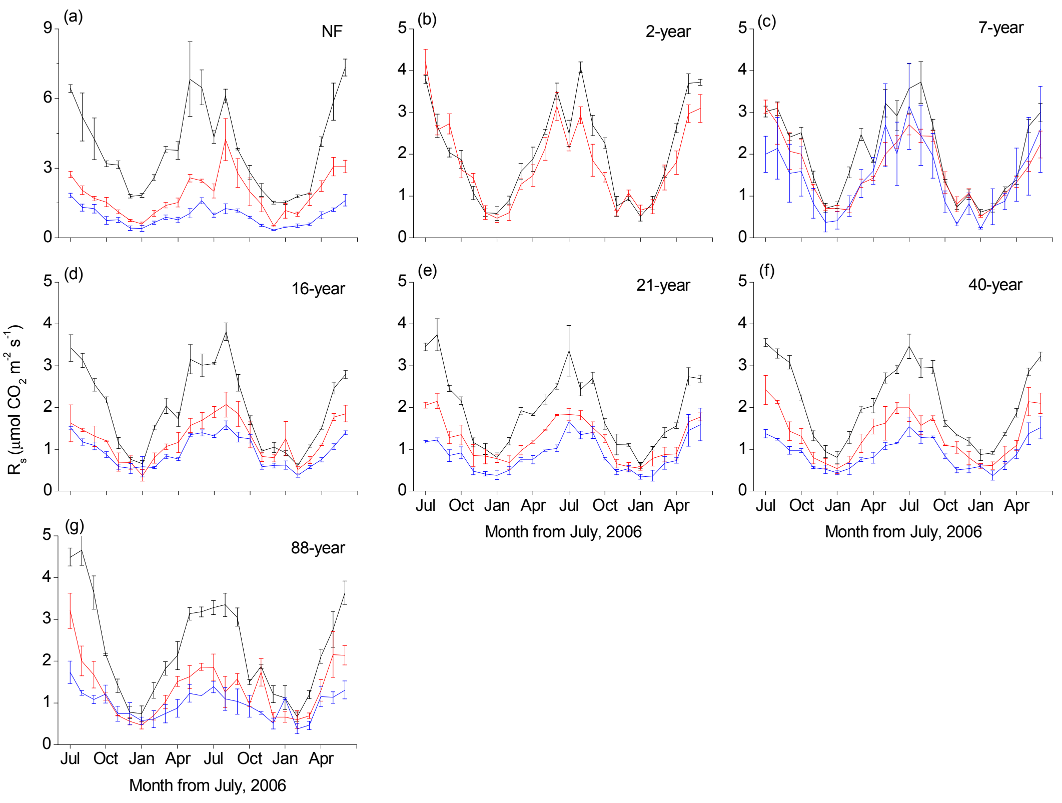

Supplementary Figure S3. Month soil respiration (R_S) rates from July 2006 to June 2008 for the intact (black), root exclusion (red), and litter plus root exclusion (blue) treatments across the Chinese fir chronosequence in southern China (from 2- to 88-year-old, native forest (NF) as the control). The error bars represent standard errors [n = 3 (plots)].

Supplementary Table S1. Main site characteristics and soil properties across the Chinese fir chronosequence in southern China (from 2- to 88-year-old, native forest (NF) as the control)

Variable	NF[1)]	2-yr	7-yr	16-yr	21-yr	40-yr	88-yr
Stem density (ha^{-1})	497	5210	4892	3875	2800	1317	750
Mean DBH (cm)	19.7	—	8.0	11.0	14.7	24.3	32.8
0-20 cm soil properties							
Total N (g kg^{-1})	1.90	1.62	1.73	1.72	1.48	1.47	1.68
Total P (g kg^{-1})	0.23	0.30	0.24	0.28	0.24	0.28	0.27
Total K (g kg^{-1})	52.3	49.4	46.1	51.0	56.1	48.7	44.6
NH_4^+-N(mg kg^{-1})	12.07	19.7	4.35	4.68	6.85	5.63	8.33
NO_3^--N(mg kg^{-1})	4.79	18.1	2.71	3.54	3.96	3.78	3.93
NH_4^+-N plus NO_3^--N (mg kg^{-1})	16.7	37.8	7.06	8.22	10.8	9.41	12.3
Available P (mg kg^{-1})	4.71	3.77	2.31	2.62	3.42	2.80	2.63
Available K (mg kg^{-1})	89.1	80.1	70.6	60.0	70.1	75.4	68.1
0-100cm SOC (t C ha^{-1})	101.2	88.7	73.0	61.8	73.0	71.4	71.9
Fine-root (<2mm diameter) biomass (g C m^{-2})	394	71.0	150	183	212	255	261
Litterfall (g C m^{-2} yr^{-1})	329	—	64	144	168	204	153

Note: 1) In the NF stand, the total stem density and the mean DBH were calculated only for the three dominant species, *Castanopsis fargesii*, *Schima superba*, and *Pinus massoniana*.

Supplementary Table S2. Results of the repeated measures ANOVA showing the *P* values for the responses of soil respiration rates (R_s), soil temperature at 5 cm soil depth (T_5), and soil moisture at 0-12 cm soil depth (W_{12}) to stand types, treatment and sampling months.

Factor	R_s	T_5	W_{12}
Month	<0.001	<0.001	<0.001
Treatment × Month	<0.001	<0.001	<0.001
Stand × Month	<0.001	<0.001	<0.001
Treatment × Stand × Month	<0.001	0.004	0.001
Treatment	<0.001	0.952	0.001
Stand	<0.001	<0.001	<0.001
Treatment × Stand	<0.001	0.023	0.900

Supplementary Table S3. Linear regression of ln-transformed soil respiration rate against soil temperature at 5 cm depth for the intact (INT), root exclusion (NR), and litter plus root exclusion (NLNR) treatments across the Chinese fir chronosequence in southern China (from 2- to 88-year-old, native forest (NF) as the control). Slopes with the same capital letters are not significantly different among stands and those with the same low-case letters are not significantly different among treatments ($p > 0.05$). CI, 95 % confidence interval; Q_{10}, temperature sensitivity, calculated from the slopes (β) as $Q_{10}=e^{10\times\beta}$. n = 24 (months)

Stands	Treatment	R^2	Slope	Lower CI	Upper CI	Intercept	Lower CI	Upper CI	Q_{10}
NF	INT	0.825	0.074Aa	0.059	0.090	-0.158	-0.455	0.139	2.10
2-yr	INT	0.894	0.078Aa	0.066	0.090	-1.135	-1.404	-0.866	2.18
7-yr	INT	0.904	0.082Aa	0.070	0.094	-0.920	-1.146	-0.694	2.27
16-yr	INT	0.935	0.077Aa	0.068	0.086	-0.892	-1.070	-0.713	2.16
21-yr	INT	0.929	0.073Aa	0.065	0.082	-0.776	-0.948	-0.604	2.08
40-yr	INT	0.959	0.078Aa	0.071	0.085	-0.802	-0.941	-0.663	2.19
88-yr	INT	0.897	0.091Aa	0.078	0.105	-1.017	-1.284	-0.750	2.49
NF	NR	0.705	0.070Aa	0.050	0.090	-0.815	-1.208	-0.421	2.02
2-yr	NR	0.842	0.072Aa	0.058	0.086	-1.132	-1.446	-0.818	2.06
7-yr	NR	0.899	0.071Aa	0.060	0.081	-0.912	-1.112	-0.712	2.03
16-yr	NR	0.733	0.052Ab	0.038	0.066	-0.858	-1.137	-0.578	1.69
21-yr	NR	0.855	0.058Aa	0.048	0.069	-0.936	-1.141	-0.731	1.79
40-yr	NR	0.929	0.069Aab	0.061	0.078	-1.102	-1.267	-0.936	2.00
88-yr	NR	0.785	0.072Aa	0.055	0.088	-1.189	-1.519	-0.859	2.04
NF	NLNR	0.839	0.069BCa	0.056	0.083	-1.474	-1.736	-1.212	2.00
2-yr	NLNR[1)]	0.842	0.072BCa	0.058	0.086	-1.132	-1.446	-0.818	2.06
7-yr	NLNR	0.826	0.095Da	0.076	0.114	-1.567	-1.939	-1.194	2.58
16-yr	NLNR	0.789	0.050Ab	0.038	0.061	-1.053	-1.277	-0.829	1.65
21-yr	NLNR	0.917	0.070BCa	0.061	0.080	-1.539	-1.720	-1.359	2.02
40-yr	NLNR	0.891	0.062ACb	0.053	0.072	-1.339	-1.527	-1.150	1.86
88-yr	NLNR	0.587	0.046Ab	0.029	0.063	-0.971	-1.310	-0.631	1.59

[1)] Using the same data of the NR treatment.

Supplementary Table S4. The multilinear regression of ln-transformed R_s against soil temperature at 5 cm depth (T_5) and soil moisture at 0-12 depths (W_{12}) or ln-transformed W_{12} for the intact (INT), root exclusion (NR), and litter plus root exclusion (NLNR) treatments across the Chinese fir chronosequence in southern China (from 2- to 88-year-old, native forest (NF) as the control). Only model resutls with significant moisture term were listed, otherwise indicated by "/". n = 24 (months)

Stands	Model		
	INT	NR	NLNR
NF	lnR_s=-2.523+0.070T_5+0.807lnW_{12}, R^2=0.937, P<0.001	/	/
2-yr	/	lnR_s=-2.780+0.070 T_5 +0.618lnW_{12}, R^2=0.881, P<0.001	/
7-yr	lnR_s=-2.123+0.079 T_5 +0.397lnW_{12}, R^2=0.918, P<0.001	/	/
16-yr	/	/	/
21-yr	/	/	/
40-yr	/	/	/
88-yr	/	/	/
